# D. M. Parker

**Published:** 1887-11

**Authors:** 


### Obituary.

---

D. M. PARKER, D. D. S.—Dr. D. M. Parker died on the 8th of October, at his residence, 132 Boylston street, Boston, of cerebral hæmorrhage, after an illness of ten days. Dr. Parker was born in Bedford, N. H., graduated in medicine when quite a young man, afterward studied dentistry in Nashua, went to Boston in 1845, and was for many years associated with the late D. Harwood, M. D., at No. 21 Summer street. For nearly twenty years he had been practicing on Boylston street, and was one of the first medical men to locate there. Dr. Parker was an earnest advocate of a thorough medical education for all dentists. He was for two years president of the American Academy of Dental Science, was a member of the Suffolk District and Massachusetts Medical Societies, was also a member of the old New England Guards, and of many other philanthropic, scientific and social organizations. In 1863 he married the eldest daughter of the late Charles Bockus, an old-time Boston merchant. His widow and one sister, Mrs. Allan Wilson, of Nashua, N. H., are the sole survivors of his immediate family. A gentle nature, universally mourned.

